# Supplementary material for: Influenza-associated excess mortality in the Philippines, 2006-2015
Source: PLoS One. 2020 Jun 17;15(6):e0234715. doi: 10.1371/journal.pone.0234715 (PMC7299398; doi:10.1371/journal.pone.0234715)
Supplement: S1 Text — (DOCX) [file pone.0234715.s001.docx]

## S1 Text. Negative binomial regression equations for each age group

For ages 0 to 4 y, and ≥60 y:

$$E\left[ Y_{t} \right]=\exp\left\{ \beta_{0}+\beta_{1}t+\beta_{2}t^{2}+\beta_{3}t^{3}+\beta_{4}t^{4}+\beta_{5}t^{5}+\beta_{6}t^{6}+ \beta_{7}\left[ Influenza A \right]_{t}+\beta_{8}\left[ Influenza B \right]_{t} + \beta_{9}\left[ \mathrm{Rainfall} \right]_{t}+\beta_{10}\left[ Mean Temperature \right]_{t}+\beta_{11}\left[ Relative Humidity \right]_{t}+\beta_{12}\left[ \mathrm{Haiyan} \right]_{t}+\beta_{13}\left[ \mathrm{Pandemic} \right]_{t}+\beta_{14}\left[ \sin\left( 2\pi t/52 \right) \right]+\beta_{15}\left[ \cos\left( 2\pi t/52 \right) \right]+\beta_{16}\left[ \sin\left( 2\pi t/26 \right) \right]+\beta_{17}\left[ \cos\left( 2\pi t/26 \right) \right]+e_{t} \right\}$$

For ages 5 to 9 y and 10 to 19 y:

$$E\left[ Y_{t} \right]=\exp\left\{ \beta_{0}+\beta_{1}t+\beta_{2}t^{2}+\beta_{3}t^{3}+\beta_{4}t^{4}+\beta_{5}t^{5}+\beta_{6}t^{6}+ \beta_{7}\left[ Influenza A \right]_{t}+\beta_{8}\left[ Influenza B \right]_{t} + \beta_{9}\left[ \mathrm{Rainfall} \right]_{t}+\beta_{10}\left[ Mean Temperature \right]_{t}+\beta_{11}\left[ Relative Humidity \right]_{t}+\beta_{12}\left[ \mathrm{Haiyan} \right]_{t}+\beta_{13}\left[ \mathrm{Pandemic} \right]_{t}+\beta_{14}\left[ \sin\left( 2\pi t/26 \right) \right]+\beta_{15}\left[ \cos\left( 2\pi t/26 \right) \right]+e_{t} \right\}$$

For ages 20 to 59 y:

$$E\left[ Y_{t} \right]=\exp\left\{ \beta_{0}+\beta_{1}t+ \beta_{2}\left[ Influenza A \right]_{t}+\beta_{3}\left[ Influenza B \right]_{t} + \beta_{4}\left[ \mathrm{Rainfall} \right]_{t}+\beta_{5}\left[ Mean Temperature \right]_{t}+\beta_{6}\left[ Relative Humidity \right]_{t}+\beta_{7}\left[ \mathrm{Haiyan} \right]_{t}+\beta_{8}\left[ \mathrm{Pandemic} \right]_{t}+\beta_{9}\left[ \sin\left( 2\pi t/52 \right) \right]+\beta_{10}\left[ \cos\left( 2\pi t/52 \right) \right]+\beta_{11}\left[ \sin\left( 2\pi t/26 \right) \right]+\beta_{12}\left[ \cos\left( 2\pi t/26 \right) \right]+e_{t} \right\}$$
