# Supplementary material for: Influenza-associated excess mortality in the Philippines, 2006-2015
Source: PLoS One. 2020 Jun 17;15(6):e0234715. doi: 10.1371/journal.pone.0234715 (PMC7299398; doi:10.1371/journal.pone.0234715)
Supplement: S1 Table — (DOCX) [file pone.0234715.s002.docx]

## S1 Table: Average weekly all-cause deaths and influenza-positive samples, 2006−2015

| **Variable** | **Mean ± SD** | **%** | **Range** |
| --- | --- | --- | --- |
| All-cause deaths per week |  |  |  |
| 0 to 4 y | 598 ± 63 | 6.3 | 403−1,118 |
| 5 to 9 y | 100 ± 25 | 1.1 | 67−509 |
| 10 to 19 y | 241 ± 32 | 2.5 | 168−714 |
| 20 to 59 y | 3,309 ± 254 | 34.8 | 2,757−5,120 |
| ≥60 y | 5,252 ± 629 | 55.3 | 3,992−7,422 |
| All ages | 9,499 ± 884 | 100.0 | 7,842−14,480 |
|  |  |  |  |
| Samples tested for influenza per week^a^ | 165 ± 216 | 100.0 | 1−2,757 |
| Samples positive for:^a^ |  |  |  |
| Influenza A & B | 28 ± 109 | 17.2 | 0−1,396 |
| Influenza A | 22 ± 109 | 13.4 | 0−1,396 |
| Influenza B | 6 ± 10 | 3.8 | 0−85 |

Abbreviation: SD, standard deviation

^a^ FluNet data missing for Week 16 of 2008
