# Supplementary material for: Influenza-associated excess mortality in the Philippines, 2006-2015
Source: PLoS One. 2020 Jun 17;15(6):e0234715. doi: 10.1371/journal.pone.0234715 (PMC7299398; doi:10.1371/journal.pone.0234715)
Supplement: S2 Table — (DOCX) [file pone.0234715.s003.docx]

## S2 Table. Model Fitting Algorithm

| Model # | Flu A | | Flu B | t1 | t2 | t3 | t4 | t5 | t6 | Rain | Temp | RH | Haiyan | Pandemic | sin(2πt/52) | cos(2πt/52) | sin(2πt/26) | cos(2πt/26) |
| --- | --- | --- | --- | --- | --- | --- | --- | --- | --- | --- | --- | --- | --- | --- | --- | --- | --- | --- |
| 1 | ✓ | | ✓ | ✓ | ✓ | ✓ | ✓ | ✓ | ✓ | ✓ | ✓ | ✓ | ✓ | ✓ | ✓ | ✓ | ✓ | ✓ |
| 2 | ✓ | | ✓ | ✓ | ✓ | ✓ | ✓ | ✓ | ✓ | ✓ | ✓ | ✓ | ✓ | ✓ | ✓ | ✓ |  |  |
| 3 | ✓ | | ✓ | ✓ | ✓ | ✓ | ✓ | ✓ | ✓ | ✓ | ✓ | ✓ | ✓ | ✓ |  |  | ✓ | ✓ |
| 4 | ✓ | | ✓ | ✓ | ✓ | ✓ | ✓ | ✓ | ✓ | ✓ | ✓ | ✓ | ✓ | ✓ |  |  | ✓ | ✓ |
| 5 | ✓ | | ✓ | ✓ | ✓ | ✓ | ✓ | ✓ |  | ✓ | ✓ | ✓ | ✓ | ✓ | ✓ | ✓ | ✓ | ✓ |
| 6 | ✓ | | ✓ | ✓ | ✓ | ✓ | ✓ | ✓ |  | ✓ | ✓ | ✓ | ✓ | ✓ | ✓ | ✓ | ✓ | ✓ |
| 7 | ✓ | | ✓ | ✓ | ✓ | ✓ | ✓ | ✓ |  | ✓ | ✓ | ✓ | ✓ | ✓ |  |  | ✓ | ✓ |
| 8 | ✓ | | ✓ | ✓ | ✓ | ✓ | ✓ | ✓ |  | ✓ | ✓ | ✓ | ✓ | ✓ |  |  |  |  |
| 9 | ✓ | | ✓ | ✓ | ✓ | ✓ | ✓ |  |  | ✓ | ✓ | ✓ | ✓ | ✓ | ✓ | ✓ | ✓ | ✓ |
| 10 | ✓ | | ✓ | ✓ | ✓ | ✓ | ✓ |  |  | ✓ | ✓ | ✓ | ✓ | ✓ | ✓ | ✓ |  |  |
| 11 | ✓ | | ✓ | ✓ | ✓ | ✓ | ✓ |  |  | ✓ | ✓ | ✓ | ✓ | ✓ |  |  | ✓ | ✓ |
| 12 | ✓ | | ✓ | ✓ | ✓ | ✓ | ✓ |  |  | ✓ | ✓ | ✓ | ✓ | ✓ |  |  |  |  |
| 13 | ✓ | | ✓ | ✓ | ✓ | ✓ |  |  |  | ✓ | ✓ | ✓ | ✓ | ✓ | ✓ | ✓ | ✓ | ✓ |
| 14 | ✓ | | ✓ | ✓ | ✓ | ✓ |  |  |  | ✓ | ✓ | ✓ | ✓ | ✓ | ✓ | ✓ |  |  |
| 15 | ✓ | | ✓ | ✓ | ✓ | ✓ |  |  |  | ✓ | ✓ | ✓ | ✓ | ✓ |  |  | ✓ | ✓ |
| 16 | ✓ | | ✓ | ✓ | ✓ | ✓ |  |  |  | ✓ | ✓ | ✓ | ✓ | ✓ |  |  |  |  |
| 17 | ✓ | | ✓ | ✓ | ✓ |  |  |  |  | ✓ | ✓ | ✓ | ✓ | ✓ | ✓ | ✓ | ✓ | ✓ |
| 18 | ✓ | | ✓ | ✓ | ✓ |  |  |  |  | ✓ | ✓ | ✓ | ✓ | ✓ | ✓ | ✓ |  |  |
| 19 | ✓ | | ✓ | ✓ | ✓ |  |  |  |  | ✓ | ✓ | ✓ | ✓ | ✓ |  |  | ✓ | ✓ |
| 20 | ✓ | | ✓ | ✓ | ✓ |  |  |  |  | ✓ | ✓ | ✓ | ✓ | ✓ |  |  |  |  |
| 21 | ✓ | | ✓ | ✓ |  |  |  |  |  | ✓ | ✓ | ✓ | ✓ | ✓ | ✓ | ✓ | ✓ | ✓ |
| 22 | ✓ | | ✓ | ✓ |  |  |  |  |  | ✓ | ✓ | ✓ | ✓ | ✓ | ✓ | ✓ |  |  |
| 23 | ✓ | | ✓ | ✓ |  |  |  |  |  | ✓ | ✓ | ✓ | ✓ | ✓ |  |  | ✓ | ✓ |
| 24 | ✓ | | ✓ | ✓ |  |  |  |  |  | ✓ | ✓ | ✓ | ✓ | ✓ |  |  |  |  |
| 25 | ✓ | | ✓ |  |  |  |  |  |  | ✓ | ✓ | ✓ | ✓ | ✓ | ✓ | ✓ | ✓ | ✓ |
| 26 | ✓ | | ✓ |  |  |  |  |  |  | ✓ | ✓ | ✓ | ✓ | ✓ | ✓ | ✓ |  |  |
| 27 | ✓ | | ✓ |  |  |  |  |  |  | ✓ | ✓ | ✓ | ✓ | ✓ |  |  | ✓ | ✓ |
| 28 | ✓ | | ✓ |  |  |  |  |  |  | ✓ | ✓ | ✓ | ✓ | ✓ |  |  |  |  |
| 29 | ✓ with 1 lag | | ✓ with 1 lag | ✓ | ✓ | ✓ | ✓ | ✓ | ✓ | ✓ | ✓ | ✓ | ✓ | ✓ | ✓ | ✓ | ✓ | ✓ |
| 30 | ✓ with 1 lag | | ✓ with 1 lag | ✓ | ✓ | ✓ | ✓ | ✓ | ✓ | ✓ | ✓ | ✓ | ✓ | ✓ | ✓ | ✓ |  |  |
| 31 | ✓ with 1 lag | | ✓ with 1 lag | ✓ | ✓ | ✓ | ✓ | ✓ | ✓ | ✓ | ✓ | ✓ | ✓ | ✓ |  |  | ✓ | ✓ |
| 32 | ✓ with 1 lag | | ✓ with 1 lag | ✓ | ✓ | ✓ | ✓ | ✓ | ✓ | ✓ | ✓ | ✓ | ✓ | ✓ |  |  | ✓ | ✓ |
| 33 | ✓ with 1 lag | | ✓ with 1 lag | ✓ | ✓ | ✓ | ✓ | ✓ |  | ✓ | ✓ | ✓ | ✓ | ✓ | ✓ | ✓ | ✓ | ✓ |
| 34 | ✓ with 1 lag | | ✓ with 1 lag | ✓ | ✓ | ✓ | ✓ | ✓ |  | ✓ | ✓ | ✓ | ✓ | ✓ | ✓ | ✓ | ✓ | ✓ |
| 35 | ✓ with 1 lag | | ✓ with 1 lag | ✓ | ✓ | ✓ | ✓ | ✓ |  | ✓ | ✓ | ✓ | ✓ | ✓ |  |  | ✓ | ✓ |
| 36 | ✓ with 1 lag | | ✓ with 1 lag | ✓ | ✓ | ✓ | ✓ | ✓ |  | ✓ | ✓ | ✓ | ✓ | ✓ |  |  |  |  |
| 37 | ✓ with 1 lag | | ✓ with 1 lag | ✓ | ✓ | ✓ | ✓ |  |  | ✓ | ✓ | ✓ | ✓ | ✓ | ✓ | ✓ | ✓ | ✓ |
| 38 | ✓ with 1 lag | | ✓ with 1 lag | ✓ | ✓ | ✓ | ✓ |  |  | ✓ | ✓ | ✓ | ✓ | ✓ | ✓ | ✓ |  |  |
| 39 | ✓ with 1 lag | | ✓ with 1 lag | ✓ | ✓ | ✓ | ✓ |  |  | ✓ | ✓ | ✓ | ✓ | ✓ |  |  | ✓ | ✓ |
| 40 | ✓ with 1 lag | | ✓ with 1 lag | ✓ | ✓ | ✓ | ✓ |  |  | ✓ | ✓ | ✓ | ✓ | ✓ |  |  |  |  |
| 41 | ✓ with 1 lag | | ✓ with 1 lag | ✓ | ✓ | ✓ |  |  |  | ✓ | ✓ | ✓ | ✓ | ✓ | ✓ | ✓ | ✓ | ✓ |
| 42 | ✓ with 1 lag | | ✓ with 1 lag | ✓ | ✓ | ✓ |  |  |  | ✓ | ✓ | ✓ | ✓ | ✓ | ✓ | ✓ |  |  |
| 43 | ✓ with 1 lag | | ✓ with 1 lag | ✓ | ✓ | ✓ |  |  |  | ✓ | ✓ | ✓ | ✓ | ✓ |  |  | ✓ | ✓ |
| 44 | ✓ with 1 lag | | ✓ with 1 lag | ✓ | ✓ | ✓ |  |  |  | ✓ | ✓ | ✓ | ✓ | ✓ |  |  |  |  |
| 45 | ✓ with 1 lag | | ✓ with 1 lag | ✓ | ✓ |  |  |  |  | ✓ | ✓ | ✓ | ✓ | ✓ | ✓ | ✓ | ✓ | ✓ |
| 46 | ✓ with 1 lag | | ✓ with 1 lag | ✓ | ✓ |  |  |  |  | ✓ | ✓ | ✓ | ✓ | ✓ | ✓ | ✓ |  |  |
| 47 | ✓ with 1 lag | | ✓ with 1 lag | ✓ | ✓ |  |  |  |  | ✓ | ✓ | ✓ | ✓ | ✓ |  |  | ✓ | ✓ |
| 48 | ✓ with 1 lag | | ✓ with 1 lag | ✓ | ✓ |  |  |  |  | ✓ | ✓ | ✓ | ✓ | ✓ |  |  |  |  |
| 49 | ✓ with 1 lag | | ✓ with 1 lag | ✓ |  |  |  |  |  | ✓ | ✓ | ✓ | ✓ | ✓ | ✓ | ✓ | ✓ | ✓ |
| 50 | ✓ with 1 lag | | ✓ with 1 lag | ✓ |  |  |  |  |  | ✓ | ✓ | ✓ | ✓ | ✓ | ✓ | ✓ |  |  |
| 51 | ✓ with 1 lag | | ✓ with 1 lag | ✓ |  |  |  |  |  | ✓ | ✓ | ✓ | ✓ | ✓ |  |  | ✓ | ✓ |
| 52 | ✓ with 1 lag | | ✓ with 1 lag | ✓ |  |  |  |  |  | ✓ | ✓ | ✓ | ✓ | ✓ |  |  |  |  |
| 53 | ✓ with 1 lag | | ✓ with 1 lag |  |  |  |  |  |  | ✓ | ✓ | ✓ | ✓ | ✓ | ✓ | ✓ | ✓ | ✓ |
| 54 | ✓ with 1 lag | | ✓ with 1 lag |  |  |  |  |  |  | ✓ | ✓ | ✓ | ✓ | ✓ | ✓ | ✓ |  |  |
| 55 | ✓ with 1 lag | | ✓ with 1 lag |  |  |  |  |  |  | ✓ | ✓ | ✓ | ✓ | ✓ |  |  | ✓ | ✓ |
| 56 | ✓ with 1 lag | | ✓ with 1 lag |  |  |  |  |  |  | ✓ | ✓ | ✓ | ✓ | ✓ |  |  |  |  |
| 57 | ✓ with 2 lags | | ✓ with 2 lags | ✓ | ✓ | ✓ | ✓ | ✓ | ✓ | ✓ | ✓ | ✓ | ✓ | ✓ | ✓ | ✓ | ✓ | ✓ |
| 58 | ✓ with 2 lags | | ✓ with 2 lags | ✓ | ✓ | ✓ | ✓ | ✓ | ✓ | ✓ | ✓ | ✓ | ✓ | ✓ | ✓ | ✓ |  |  |
| 59 | ✓ with 2 lags | ✓ with 2 lags | | ✓ | ✓ | ✓ | ✓ | ✓ | ✓ | ✓ | ✓ | ✓ | ✓ | ✓ |  |  | ✓ | ✓ |
| 60 | ✓ with 2 lags | ✓ with 2 lags | | ✓ | ✓ | ✓ | ✓ | ✓ | ✓ | ✓ | ✓ | ✓ | ✓ | ✓ |  |  | ✓ | ✓ |
| 61 | ✓ with 2 lags | ✓ with 2 lags | | ✓ | ✓ | ✓ | ✓ | ✓ |  | ✓ | ✓ | ✓ | ✓ | ✓ | ✓ | ✓ | ✓ | ✓ |
| 62 | ✓ with 2 lags | ✓ with 2 lags | | ✓ | ✓ | ✓ | ✓ | ✓ |  | ✓ | ✓ | ✓ | ✓ | ✓ | ✓ | ✓ | ✓ | ✓ |
| 63 | ✓ with 2 lags | ✓ with 2 lags | | ✓ | ✓ | ✓ | ✓ | ✓ |  | ✓ | ✓ | ✓ | ✓ | ✓ |  |  | ✓ | ✓ |
| 64 | ✓ with 2 lags | ✓ with 2 lags | | ✓ | ✓ | ✓ | ✓ | ✓ |  | ✓ | ✓ | ✓ | ✓ | ✓ |  |  |  |  |
| 65 | ✓ with 2 lags | ✓ with 2 lags | | ✓ | ✓ | ✓ | ✓ |  |  | ✓ | ✓ | ✓ | ✓ | ✓ | ✓ | ✓ | ✓ | ✓ |
| 66 | ✓ with 2 lags | ✓ with 2 lags | | ✓ | ✓ | ✓ | ✓ |  |  | ✓ | ✓ | ✓ | ✓ | ✓ | ✓ | ✓ |  |  |
| 67 | ✓ with 2 lags | ✓ with 2 lags | | ✓ | ✓ | ✓ | ✓ |  |  | ✓ | ✓ | ✓ | ✓ | ✓ |  |  | ✓ | ✓ |
| 68 | ✓ with 2 lags | ✓ with 2 lags | | ✓ | ✓ | ✓ | ✓ |  |  | ✓ | ✓ | ✓ | ✓ | ✓ |  |  |  |  |
| 69 | ✓ with 2 lags | ✓ with 2 lags | | ✓ | ✓ | ✓ |  |  |  | ✓ | ✓ | ✓ | ✓ | ✓ | ✓ | ✓ | ✓ | ✓ |
| 70 | ✓ with 2 lags | ✓ with 2 lags | | ✓ | ✓ | ✓ |  |  |  | ✓ | ✓ | ✓ | ✓ | ✓ | ✓ | ✓ |  |  |
| 71 | ✓ with 2 lags | ✓ with 2 lags | | ✓ | ✓ | ✓ |  |  |  | ✓ | ✓ | ✓ | ✓ | ✓ |  |  | ✓ | ✓ |
| 72 | ✓ with 2 lags | ✓ with 2 lags | | ✓ | ✓ | ✓ |  |  |  | ✓ | ✓ | ✓ | ✓ | ✓ |  |  |  |  |
| 73 | ✓ with 2 lags | ✓ with 2 lags | | ✓ | ✓ |  |  |  |  | ✓ | ✓ | ✓ | ✓ | ✓ | ✓ | ✓ | ✓ | ✓ |
| 74 | ✓ with 2 lags | ✓ with 2 lags | | ✓ | ✓ |  |  |  |  | ✓ | ✓ | ✓ | ✓ | ✓ | ✓ | ✓ |  |  |
| 75 | ✓ with 2 lags | ✓ with 2 lags | | ✓ | ✓ |  |  |  |  | ✓ | ✓ | ✓ | ✓ | ✓ |  |  | ✓ | ✓ |
| 76 | ✓ with 2 lags | ✓ with 2 lags | | ✓ | ✓ |  |  |  |  | ✓ | ✓ | ✓ | ✓ | ✓ |  |  |  |  |
| 77 | ✓ with 2 lags | ✓ with 2 lags | | ✓ |  |  |  |  |  | ✓ | ✓ | ✓ | ✓ | ✓ | ✓ | ✓ | ✓ | ✓ |
| 78 | ✓ with 2 lags | ✓ with 2 lags | | ✓ |  |  |  |  |  | ✓ | ✓ | ✓ | ✓ | ✓ | ✓ | ✓ |  |  |
| 79 | ✓ with 2 lags | ✓ with 2 lags | | ✓ |  |  |  |  |  | ✓ | ✓ | ✓ | ✓ | ✓ |  |  | ✓ | ✓ |
| 80 | ✓ with 2 lags | ✓ with 2 lags | | ✓ |  |  |  |  |  | ✓ | ✓ | ✓ | ✓ | ✓ |  |  |  |  |
| 81 | ✓ with 2 lags | ✓ with 2 lags | |  |  |  |  |  |  | ✓ | ✓ | ✓ | ✓ | ✓ | ✓ | ✓ | ✓ | ✓ |
| 82 | ✓ with 2 lags | ✓ with 2 lags | |  |  |  |  |  |  | ✓ | ✓ | ✓ | ✓ | ✓ | ✓ | ✓ |  |  |
| 83 | ✓ with 2 lags | ✓ with 2 lags | |  |  |  |  |  |  | ✓ | ✓ | ✓ | ✓ | ✓ |  |  | ✓ | ✓ |
| 84 | ✓ with 2 lags | ✓ with 2 lags | |  |  |  |  |  |  | ✓ | ✓ | ✓ | ✓ | ✓ |  |  |  |  |

*Note: Flu A and Flu B are percentage of samples confirmed positive for influenza A and influenza B, respectively; t1 to t6 are the polynomial time trends; Rain, Temp, and RH pertain to rainfall, weekly average temperature, and relative humidity, respectively; sin(2πt/52) and cos(2πt/52) are the annual cyclical terms; and sin(2πt/26) and cos(2πt/26) are the semiannual cyclical terms.
