# Supplementary material for: Influenza-associated excess mortality in the Philippines, 2006-2015
Source: PLoS One. 2020 Jun 17;15(6):e0234715. doi: 10.1371/journal.pone.0234715 (PMC7299398; doi:10.1371/journal.pone.0234715)
Supplement: S3 Table — (DOCX) [file pone.0234715.s004.docx]

## S3 Table. Selected negative binomial regression models

|  | **Age group** | | | | |
| --- | --- | --- | --- | --- | --- |
| **Independent variable** | **0 to 4 y** | **5 to 9 y** | **10 to 19 y** | **20 to 59 y** | **≥60 y** |
| **Time trends** |  |  |  |  |  |
| **t** | -0.0001 | -0.0012** | -0.0012** | 0.0004*** | 0.0008*** |
|  | (0.0003) | (0.0006) | (0.0006) | (0.0000) | (0.0002) |
| **t^2^** | 0.0000 | 0.0000 | 0.0000 |  | -0.0000 |
|  | (0.0000) | (0.0000) | (0.0000) |  | (0.0000) |
| **t^3^** | 0.0000* | 0.0000 | 0.0000 |  | 0.0000 |
|  | (0.0000) | (0.0000) | (0.0000) |  | (0.0000) |
| **t^4^** | -0.0000** | -0.0000** | -0.0000** |  | 0.0000 |
|  | (0.0000) | (0.0000) | (0.0000) |  | (0.0000) |
| **t^5^** | 0.0000 | -0.0000 | -0.0000 |  | -0.0000 |
|  | (0.0000) | (0.0000) | (0.0000) |  | (0.0000) |
| **t^6^** | 0.0000 | 0.0000** | 0.0000** |  | -0.0000 |
|  | (0.0000) | (0.0000) | (0.0000) |  | (0.0000) |
| **% Influenza A-to-total samples tested** |  |  |  |  |  |
| **Current period** | 0.0520 | 0.0432 | 0.0691 | 0.0607*** | 0.0776*** |
|  | (0.0475) | (0.0904) | (0.0561) | (0.0203) | (0.0290) |
| **First Lag** | 0.0105 | 0.1941** | -0.0466 | 0.0033 | 0.0353 |
|  | (0.0500) | (0.0966) | (0.0603) | (0.0216) | (0.0307) |
| **Second Lag** | 0.0212 | 0.0694 | 0.0710 | 0.0197 | 0.0409 |
|  | (0.0464) | (0.0896) | (0.0558) | (0.0201) | (0.0286) |
| **% Influenza B-to-total samples tested** |  |  |  |  |  |
| **Current period** | 0.1445 | 0.2409 | -0.0340 | 0.0285 | 0.0856 |
|  | (0.0944) | (0.1803) | (0.1136) | (0.0405) | (0.0578) |
| **First Lag** | -0.0692 | -0.0732 | -0.0086 | -0.0050 | -0.0176 |
|  | (0.1014) | (0.1942) | (0.1218) | (0.0436) | (0.0621) |
| **Second Lag** | -0.0277 | 0.0892 | 0.0737 | -0.0197 | -0.0208 |
|  | (0.0968) | (0.1850) | (0.1166) | (0.0417) | (0.0595) |
| **Meteorological controls** |  |  |  |  |  |
| **Rainfall** | -0.0011 | 0.0024 | 0.0025** | 0.0008* | 0.0000 |
|  | (0.0011) | (0.0020) | (0.0013) | (0.0005) | (0.0007) |
| **Weekly mean temperature** | 0.0392*** | 0.0517*** | 0.0304*** | 0.0095*** | -0.0104** |
|  | (0.0067) | (0.0108) | (0.0067) | (0.0029) | (0.0041) |
| **Relative humidity** | 0.0053** | -0.0024 | -0.0065** | -0.0047*** | -0.0044*** |
|  | (0.0025) | (0.0047) | (0.0029) | (0.0010) | (0.0015) |
| **Annual seasonality** |  |  |  |  |  |
| **Sine** | -0.0754*** | -0.0334*** | -0.0312*** | -0.0162*** | -0.0143*** |
|  | (0.0063) | (0.0116) | (0.0073) | (0.0026) | (0.0039) |
| **Cosine** | 0.0289*** | 0.0212 | 0.0292*** | 0.0157*** | 0.0289*** |
|  | (0.0087) | (0.0156) | (0.0098) | (0.0038) | (0.0054) |
| **Semiannual seasonality** |  |  |  |  |  |
| **Sine** | 0.0239*** |  |  | -0.0049** | 0.0057* |
|  | (0.0050) |  |  | (0.0022) | (0.0031) |
| **Cosine** | 0.0285*** |  |  | 0.0128*** | 0.0112*** |
|  | (0.0059) |  |  | (0.0025) | (0.0036) |
| **Other Controls** |  |  |  |  |  |
| **Typhoon Haiyan (1=Yes)** | -0.0008 | 0.0059 | 0.0232 | 0.0083 | 0.0090 |
|  | (0.0135) | (0.0254) | (0.0159) | (0.0053) | (0.0083) |
| **2009 Pandemic (1=Yes)** | 0.6063*** | 1.6378*** | 1.0846*** | 0.3720*** | 0.1739*** |
|  | (0.0721) | (0.1157) | (0.0759) | (0.0318) | (0.0470) |
| **Constant** | 4.8737*** | 3.4181*** | 5.1470*** | 8.1094*** | 9.0201*** |
|  | (0.2831) | (0.5233) | (0.3275) | (0.1193) | (0.1727) |
| **Akaike information criterion** | 5,345.10 | 4,145.9 | 4,589.63 | 6,236.66 | 7,057.59 |

*, **, and *** indicate 1%, 5%, and 10% significance level, respectively. Standard errors are given in parentheses.
