# Supplementary material for: Influenza-associated excess mortality in the Philippines, 2006-2015
Source: PLoS One. 2020 Jun 17;15(6):e0234715. doi: 10.1371/journal.pone.0234715 (PMC7299398; doi:10.1371/journal.pone.0234715)
Supplement: S4 Table — (DOCX) [file pone.0234715.s005.docx]

## S4 Table. Sensitivity analyses

|  | **Original result** | **Analysis A** | **Analysis B** | **Analysis C** | **Analysis D** |
| --- | --- | --- | --- | --- | --- |
| **Modifications** |  |  |  |  |  |
| Imputed all-cause deaths for week 45 of 2013^a^ | − | No | No | Yes | Yes |
| Imputed influenza positivity rate for weeks with ≤1 sample tested | − | Yes | Yes | Yes | Yes |
| Imputed influenza positivity rate for weeks with ≤10 samples tested | − | No | Yes | No | Yes |
|  |  |  |  |  |  |
| **Average annual EMR (95% CI)** |  |  |  |  |  |
| 0 to 4 y | 2.14 (0.44–2.19) | 2.04 (1.98–2.10) | 2.19 (2.13–2.25) | 2.03 (1.97–2.09) | 2.18 (2.12–2.24) |
| 5 to 9 y | 1.51 (1.20–1.56) | 1.55 (1.49–1.61) | 1.50 (1.44–1.56) | 1.51 (1.45–1.57) | 1.46 (1.40–1.52) |
| 10 to 19 y | 0.48 (0.10–0.50) | 0.44 (0.41–0.48) | 0.39 (0.36–0.43) | 0.44 (0.41–0.47) | 0.39 (0.36–0.42) |
| 20 to 59 y | 2.02 (2.00–2.03) | 1.95 (1.94–1.96) | 1.72 (1.71–1.73) | 1.94 (1.93–1.96) | 1.72 (1.70–1.73) |
| ≥60 y | 44.63 (44.51–44.69) | 46.31 (46.19−46.38) | 44.92 (44.81–44.99) | 46.27 (46.15–46.34) | 44.89 (44.77–44.96) |
| All ages | 5.09 (2.20–5.09) | 5.19 (5.19–5.20) | 4.96 (4.95–4.96) | 5.19 (5.18–5.19) | 4.95 (4.94–4.95) |

Abbreviations: EMR, excess mortality rate per 100,000 individuals; CI, confidence interval

^a^ We did not include the Typhoon Haiyan week for models with this modification.
