# Supplementary figures and images for: Influenza-associated excess mortality in the Philippines, 2006-2015
Source: PLoS One. 2020 Jun 17;15(6):e0234715. doi: 10.1371/journal.pone.0234715 (PMC7299398; doi:10.1371/journal.pone.0234715)

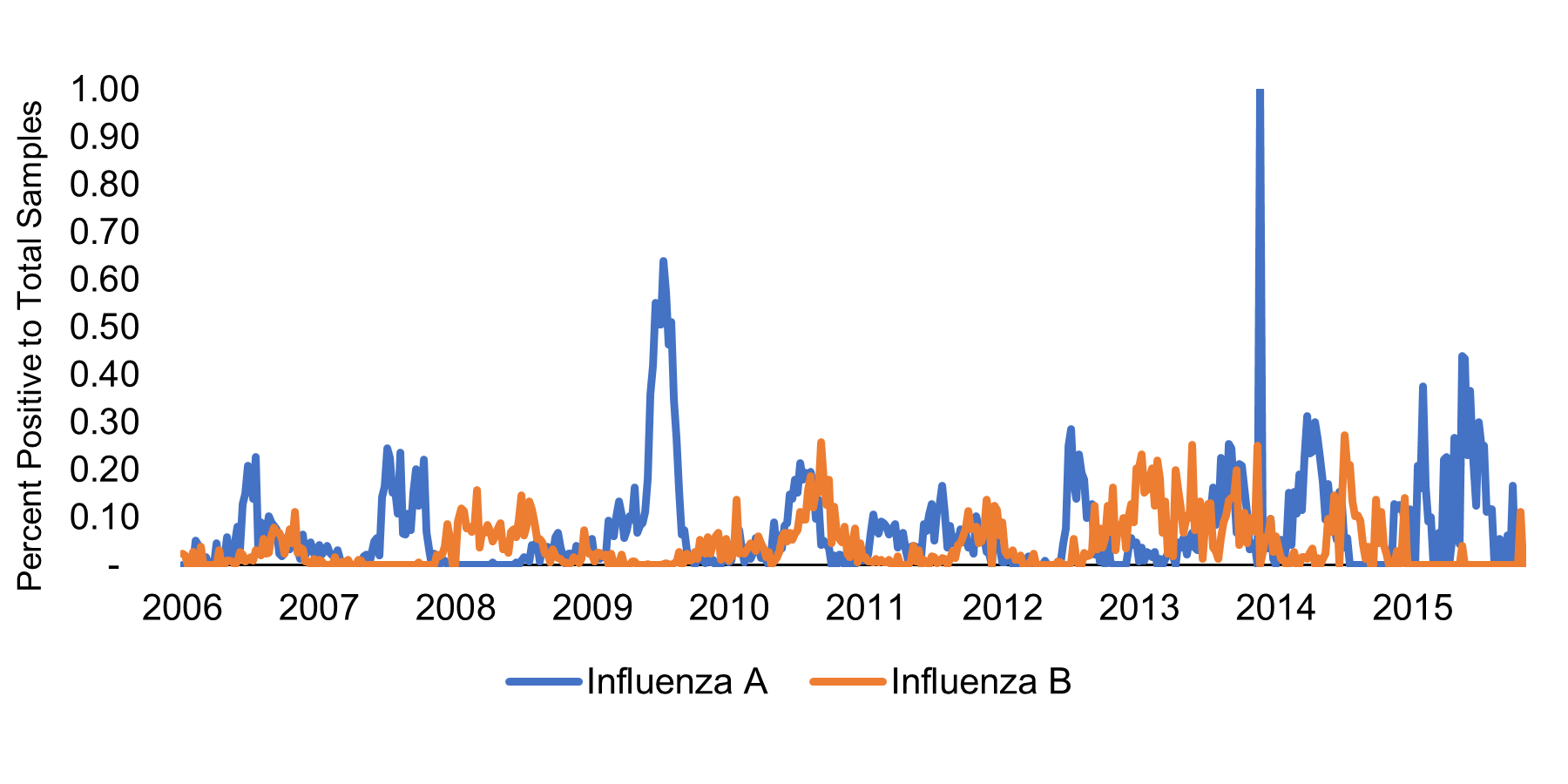

Supplement: S1 Fig — (TIF) [file pone.0234715.s006.TIF]

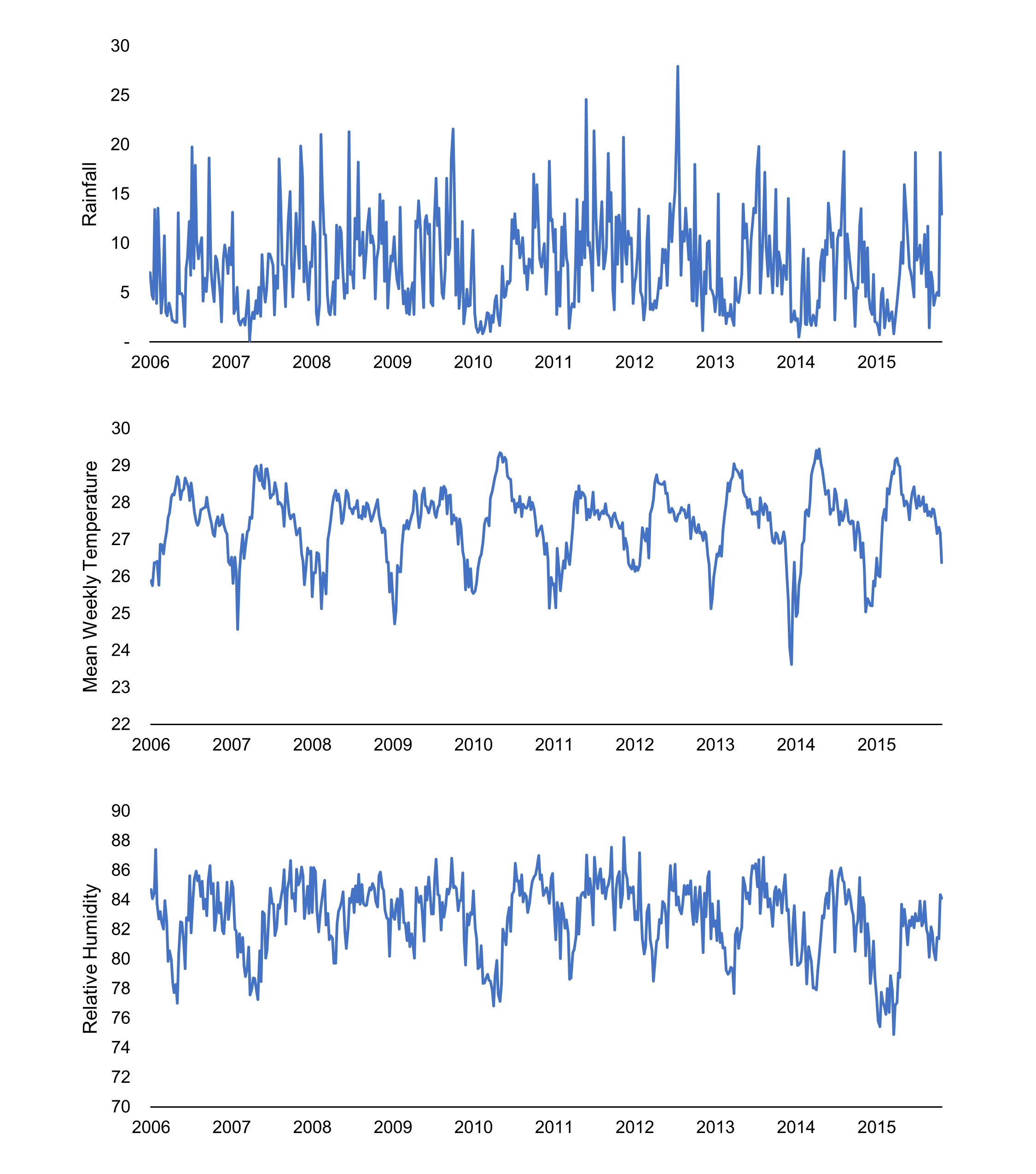

Supplement: S2 Fig — (TIF) [file pone.0234715.s007.tif]

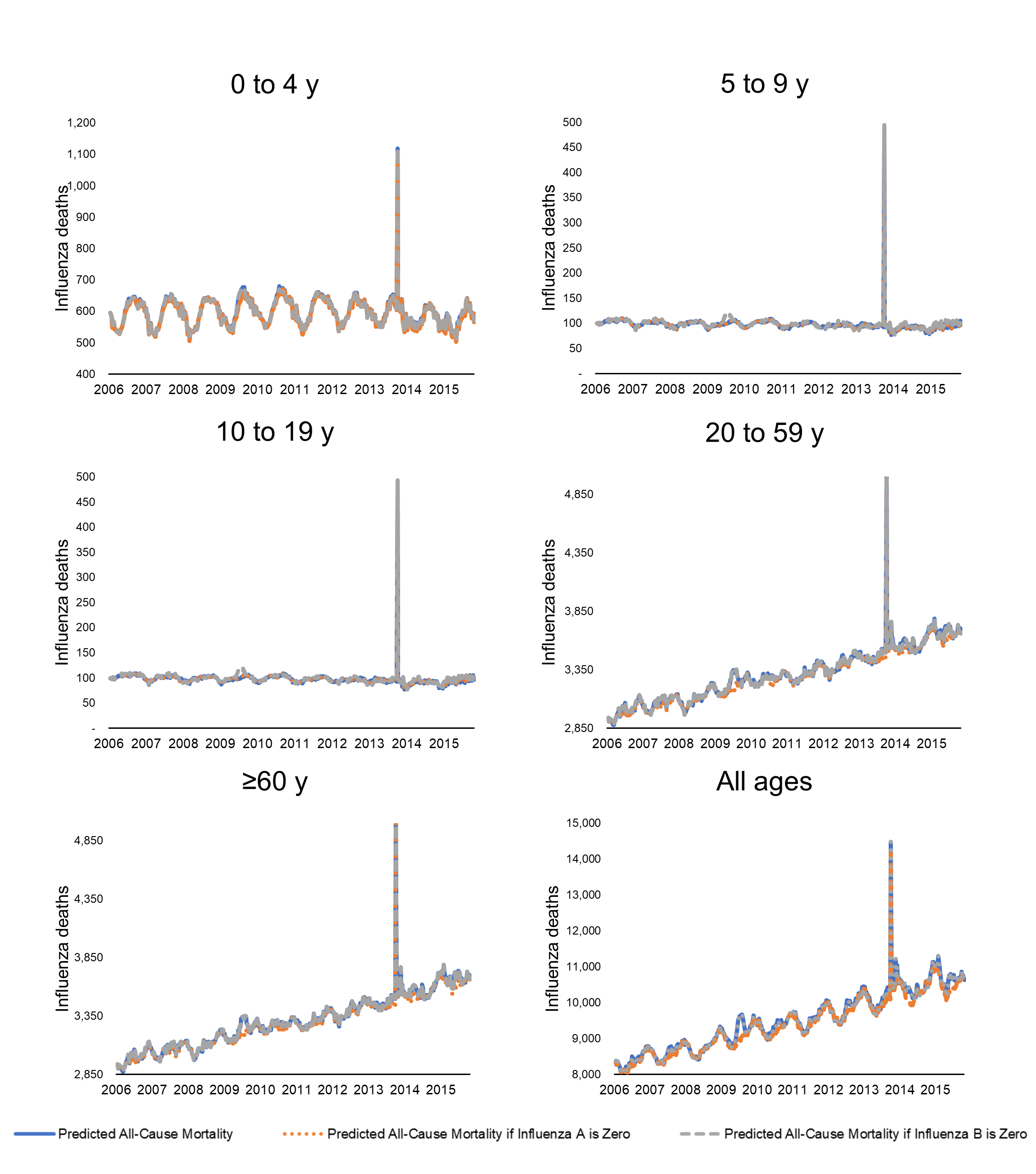

Supplement: S3 Fig — (TIF) [file pone.0234715.s008.tif]
