# Supplementary material for: Influenza-associated excess mortality in the Philippines, 2006-2015
Source: PLoS One. 2020 Jun 17;15(6):e0234715. doi: 10.1371/journal.pone.0234715 (PMC7299398; doi:10.1371/journal.pone.0234715)
Supplement: Attachment — Submitted filename: PONE-D-1924478-Review Comments.pdf [file pone.0234715.s009.pdf]

PONE-D-19-24478: Influenza-associated excess mortality in the Philippines, 2006-2015

This manuscript estimates Influenza-associated excess mortality in the Philippines during the period 2006 through 2015 using negative binomial regression models. As the authors indicate, mortality burden of influenza in the Philippines has not been quantified before thus findings from this manuscript will help to inform public health policies and strategies for the control of influenza.

Here are some comments that the authors could consider to help the readers to better understand their study and the findings that they present.

1. In line 78: The authors state, “No adjustment was made for under-registration of deaths”. Before that, the authors indicate that deaths “must be registered within 48 hours”. Isn’t this a contradiction? It would be of help to the readers if the authors could state why no adjustments were made for under-registration. Is it that no data are available to quantify deaths that are not registered, or perhaps that there is so much variation by site/hospital or region in the country to allow for meaningful adjustments?

2. The authors indicate that GISRS data were collected through ILI and SARI cases at sentinel sites. Can they include the case definitions used for ILI and SARI or provide appropriate references for the readers?

3. I note that the authors used a weekly time-series to model the overall and age-specific EMRs associated with influenza. Did the authors consider using time-lagged independent variables (particularly for meteorological variables and influenza activity)? This is not stated in the text and one would expect a time-lagged effect of these variables on mortality.

4. Also, is there a particular reason why the meteorological variables (rainfall, temperature, and humidity) were included in the model to estimate EMRS? Has there been data to suggest that these are important for the Philippines? Did inclusion of these variables result in better fitting models, across the age groups assessed?

5. In lines 118-119, the authors state the “Other missing data were not replaced in the principal analyses”. This is somehow ambiguous. Could the authors state what these data are?

6. Reading through the methods, I assume that the data from the 2009 pandemic period were included in the analyses, is this correct? If so, I would suggest that the authors rerun the analyses with the pandemic data excluded. This is particularly important if they are seeking to estimate the mean annual EMRs associated with influenza. A quick look at the data suggests an additional few hundred deaths (~300 deaths) annually if the data from the pandemic period are included.

7. Related to that, I suggest that the authors only model the estimates using the imputed deaths for week 45 in 2013 (when the typhoon Haiyan occurred). Regardless of the fact that they conducted sensitivity analyses, it is clear that the spike in deaths during that week was out of the ordinary and thus it would only make sense if it were excluded.

8. The authors mention that the 95% CIs were estimated using bootstrapping methods with 1,500 iterations. However, I note that the CIs are very narrow, particularly for data among young children <5 years. Could the authors look again at this, and perhaps comment about it in the discussion?

9. In lines 208-209, the authors state "…. and influenza was the cause of approximately one in every 100 deaths". This is not explicit in the results section but I suppose that this is based on dividing the total mean annual all-cause deaths by the mean annual influenza-associated deaths. Could the authors try to make this more explicit in the text? This further highlights why you should not include the 2009 pandemic data in your EMRs models.
