# Supplementary material for: Influenza-associated excess mortality in the Philippines, 2006-2015
Source: PLoS One. 2020 Jun 17;15(6):e0234715. doi: 10.1371/journal.pone.0234715 (PMC7299398; doi:10.1371/journal.pone.0234715)
Supplement: Attachment — Submitted filename: Phillippines review.docx [file pone.0234715.s010.docx]

The authors intend to quantify mortality associated to influenza in the Philippines, which is very important for public health and prevention. Hence, this is a very important paper.

Some general comments:

The authors use three metrological measures, rain, temperature and humidity, which each also have seasonal variation. Therefore, the inclusion of the yearly and half-yearly sines must have been included to adjust for residual seasonality not covered by these metrological variables.

Argue why a negative binomial regression (compensate for over-dispersion – what about under-dispersion?)
Additive or multiplicative model? – link function

Selection of elements to be included in the model is based on having positive A and B coefficients – argue why - and secondly the lowest AIC. However, in the S2 Table I miss this information.

Figure 2 show huge peaks in number of deaths associated to influenza in week 45 2013 (The typhoon Haiyan), This I don’t understand.

- Was there a huge peak in positive influenza samples that week? – I do not believe so, probably none or very few samples were sampled in that week.
- As there are no peaks in the model (red line) in figure 1, and the number of deaths associated to influenza was calculated as the prediction from the full model minus the prediction by the same model, but with A and B set to zero. There should not be calculated peaks.
  This indicate that the calculation of influenza-associated number of deaths is wrong!

Would be nice to show both the full models and the models with A and/or B as zero in graph 1.

Suggest including graphs showing the A and B positive percentages over calendar time used in the model and as supplementary the metrological parameters.

The authors intend to compare all-cause influenza-associated mortality with cause-specific influenza-associated mortality. The most commonly used cause-specific mortality is respiratory (ICD10 …) e.g. references 1 and 2. The authors only look at J10 and J11 (influenza the main cause of deaths). This is of cause interesting, but it would have been of more interest, if they (also) had looked at respiratory cause of deaths, and made serious comparisons, for example if there is a general relation e.g. has it been suggested that influenza-associated mortality estimated using all-cause is the double of respiratory.
Alternatively, the authors could leave out cause-specific influenza-associated mortality, and write another article comparing all-cause and cause-specific.

Suggest to include a typhoon Haiyan parameter in the model: 1 in week 45 2013, else 0.

The Philippines consist of many islands with varying population. The metrological stations are properly distributed more-or-less evenly over the whole area, why average metrological measures should be population weighted. Likewise for the influenza data.
Is this possible? – if not, this should be discussed as a limitation.

Minor comments:

Page 3, line 64: I believe ‘mortality has not been accurately quantified’ attribute too much faith in the model. Suggest to leave out ‘accurately’.

Page 4, line 66-67: ‘… and compare …’. Suggest ‘… and compare all-cause estimates with influence cause-specific estimates’.

Page 4, line 78: Is it correct that all deaths in the Philippines are registered with cause of deaths within 48 hours? – Faster than in any other country, I know of.

Page 4, line 81-86: What do you mean by and what is the difference between ILI sentinel sites and SARI (Severe acute respiratory infections) sites? – how many of each?

Page 5, line 93. I cannot find ‘average weekly temperature’ in reference 13, only average daily min and max temperatures.

Page 5, line 105: Ok to use a polynomial spline, but why 6?

Page 6, line 118: ‘Other missing data …’ - how many?

Page 13, line 212-215. I believe it is highly surprising that this studies all-cause influenza-associated mortality estimates are consistent with the respiratory-cause-specific estimates from Iuliano et al. study

Page 13, line 223: I would not use the word ‘accurately’, but something like our model fitted data well

Page 13, line 223-4: Sometimes ‘official deaths registry statistics’ stand for cause-specific and here for all-cause. Suggest using all-cause and cause-specific.

Page 13, line 224: It is not correct that your model predicted the typhoon Haiyan peak! – see figure 1

Page 14-15, line 252-255: You might have used, what is often called the Goldstein index: ILI-rate * positive-percentage, where the ILI rate reflect the population dynamics and the positive-percentage limit the ILI-rate to influenza i.e. exclude other circulating respiratory pathogens.
